# The distribution and host utilization of scleractinian coral-infesting Ascothoracida (Crustacea, Thecostraca), including the discovery of a new species of *Petrarca* from Dongsha Island, Taiwan

**DOI:** 10.3897/zookeys.1284.189493

**Published:** 2026-07-03

**Authors:** Gregory A. Kolbasov, Benny K. K. Chan

**Affiliations:** 1 White Sea Biological Station, Biological Faculty, Moscow State University, Moscow 119991, Russia White Sea Biological Station, Biological Faculty, Moscow State University Moscow Russia; 2 Biodiversity Research Center, Academia Sinica, Taipei 11529, Taiwan Biodiversity Research Center, Academia Sinica Taipei Taiwan

**Keywords:** Ascothoracida, distribution, morphology, parasitic crustaceans, Petrarcidae, SEM, stony corals, taxonomy

## Abstract

*Petrarca* is a genus of specialized crustacean ascothoracidan endoparasites that inhabit scleractinian corals and form galls on the corals’ surface. In the present study, we identified a new species, *Petrarca chengi***sp. nov**., within external galls on the deepwater coral *Deltocyathus magnificus* collected off Dongsha Island, Taiwan. We provide a description of the new species based on light and scanning electron microscopy. This species is diagnosed using the morphology of the carapace, mouthparts, and penis. Diagnostic morphological characters for *Petrarca* include: carapace shape, the morphology of carapace papillae and spines, the structure of the rudimentary abdomen, the shape of the distal part of the penis, the morphology of thoracopod 1, the cutting margins of the mandibles and maxillules, and the ultrastructure of the distal part of the aesthetasc of the antennular claw guard. The study also compares host specificity interactions among members of the family Petrarcidae, including the genera *Introcornia*, *Petrarca*, and *Zibrowia*, offering new insights into the biology of these parasitic crustaceans. We provide a comprehensive summary of existing records for these genera; the resulting data on localities, depths, and host taxonomy reveal the parasites’ vast geographical ranges and diverse host associations.

## Introduction

The subclass Ascothoracida, belonging to the crustacean class Thecostraca, comprises exclusively parasitic species that exhibit a range of parasitic strategies, from ecto- and mesoparasitism to endoparasitism. These crustaceans infest invertebrate hosts from the phyla Cnidaria (specifically the subphylum Anthozoa) and Echinodermata (Chan et al. 2021). Among cnidarians, ascothoracidans parasitize members of the anthozoan classes Octocorallia (Malacalcyonacea, Scleralcyonacea) and Hexacorallia (Antipatharia, Scleractinia, and Zoantharia). Echinoderm hosts include species belonging to the classes Asteroidea, Crinoidea, Echinoidea, and Ophiuroidea. Currently, the subclass comprises approximately 124 described species (Kolbasov et al. 2025), which are grouped into two orders, Laurida and Dendrogastrida (Grygier 1987a, 1996). Members of the order Laurida parasitize anthozoans, with the exception of the genus *Waginella* Grygier, 1983, whose species are ectoparasites of crinoids. In contrast, species of the order Dendrogastrida parasitize non-crinoid echinoderms (Kolbasov et al. 2021).

Morphologically, ascothoracidans are characterized by a bivalved carapace enclosing the body and containing gonads and gut diverticula (Chan et al. 2021). In females of more derived forms, the two valves of the carapace are partially fused and may become elaborately modified into ramified or otherwise bizarre shapes. In the generalized body plan, which may exhibit substantial modifications depending on the degree of adaptation to parasitism, the trunk consists of 11 segments (somites): six thoracic segments bearing biramous, natatory thoracopods, and five well-developed abdominal segments, including the telson, which carries a pair of unsegmented furcal rami. The first abdominal segment bears a developed penis in males or hermaphrodites, a conspicuous vestige of which persists in females. The antennules are prehensile to subchelate and typically comprise four to six segments, with a claw-like finger developed on the distal segment. The labrum surrounds the mouthparts, forming an oral cone or pyramid. Ascothoracidans generally possess three pairs of piercing mouthparts, the mandibles, maxillules, and maxillae, although this number is often reduced, and the appendages may be rudimentary in more advanced parasitic species (Grygier 1987a; Høeg et al. 2014; Kolbasov et al. 2021).

The life cycle of ascothoracidans varies among species and may include either free-swimming naupliar larvae or larvae brooded within the mantle cavity of the adult female. It potentially comprises up to six planktotrophic or lecithotrophic naupliar instars, although in some species the naupliar phase is highly condensed or entirely absent (Høeg et al. 2014). The naupliar phase is succeeded by one or two instars of a specialized, non-feeding cypridiform ascothoracid larva, which is responsible for host infection. Ascothoracidans are generally dioecious, with females being larger than males; in some taxa, males are dwarf and cypridiform in morphology (Grygier and Fratt 1984; Grygier 1985, 1987b, 1991a, 1991b; Kolbasov 2007).

The family Petrarcidae comprises endoparasitic species inhabiting scleractinian corals. Members of this family have secondarily evolved hermaphroditism and represent one of the most advanced lineages within the order Laurida (Okada 1938; Grygier 1983a, 1983b, 1983c; Kolbasov et al. 2023a). Adults are characterized by a bivalved carapace with thick, inflated valves armed with papillae and lacking a brood chamber. The antennules are five-segmented and weakly armed, while the mouth appendages are non-piercing, and the thoracopods are uniramous (Grygier 1987a). To date, 16 species have been assigned to three genera: *Petrarca* Fowler, 1889 (10 species), *Introcornia* Grygier, 1983 (2 species), and *Zibrowia* Grygier, 1985 (4 species). These parasites infect various species of azooxanthellate and zooxanthellate scleractinian corals belonging to the families Dendrophylliidae, Fungiacyathidae, Flabellidae, Madreporidae, and Caryophylliidae, which occur in shallow to deep waters (Cairns 2001; Arrigoni et al. 2014). Typically, petrarcids occur in pairs within a single spongy gall that lacks an external aperture (Kolbasov et al. 2023a, 2025). Unlike other ascothoracidans, petrarcids do not brood their embryos between the carapace valves (Grygier 1985).

*Petrarca* was established by Fowler (1889) for the abyssal species, described off the coast of Japan, from a depth of 4200 m and found in a solitary coral *Fungiacyathus marenzelleri* (Vaughan, 1906). This species was also re-described by Grygier (1985) from East Africa and north-eastern Australia. Subsequently, nine congeners were described from various locations and depths, and in different hosts (Kolbasov et al. 2023a; Tables 1, 2). In the present study, five specimens of a new species of *Petrarca* were discovered within external galls on the deepwater coral *Deltocyathus magnificus* Moseley, 1876, collected off Dongsha Island, Taiwan. In addition to previous descriptions (Kolbasov et al. 2023a), our study involves both light and scanning electron microscopy (SEM) to document the fine-scale functional external morphology of *Petrarca*. This study not only expands the biodiversity of the genus *Petrarca* but also highlights its morphological characters and distribution of the family Petrarcidae.

**Table 1.** Main diagnostic characters of species of the genus *Petrarca*.

Characters	Size of holotype (long–wide–high, mm)	Form of carapace	Papillae/spines of carapace	Terminal part of rudimentary abdomen	Distal part of penis	Thoracopod 1	Cutting margin of mandible	Cutting margin of maxillule	Claw guard antennular aesthetasc
Species									
*P. azorica*	3.3–2.8–2.9	irregularly ellipsoidal (dome–shaped), lacking lumpy inflations and radial ridges	large projections and bumps ventrally, small papillae and large spines anterolaterally	with terminal setae/spines	anterior lobes reduced, small rectangular posterior rami	not found	with many small teeth, upper fused together, lower bifid and separate	with many small teeth, upper fused together, lower separate	with simple tip (not studied in details)
*P. bathyactidis*	2.7–?–2.3 (no information on holotype)	dome–shaped, lacking lumpy inflations and radial ridges	large spines/papillae ventrally, small papillae and spines laterally	with terminal setae/spines	anterior lobes small, distinct triangular posterior rami	long setiform, with 2 short distal setae	not described	not described	with simple tip (not studied in details)
*P. chengi*	2.0–1.9–1.8	dome–shaped, lacking lumpy inflations and radial ridges	numerous small papillae ventrally, large conical papillae laterally	without terminal setae/spines	anterior lobes rounded, developed, posterior rami conspicuous, rectangular	long setiform, with simple distal end	with 10 upper long, simple teeth, 4 lower bifid teeth, lower angle with 4 sharp denticles	uneven, saw–like	with simple tip, without outgrowths
*P. goanna*	5.9–?–5.1	roughly ovoid/spherical, with radial ridges with lumpy inflations	few papillae ventrally, lateral surface without small papillae	with or without tiny terminal spines	anterior lobes reduced, small, squarish posterior rami	long setiform, with simple distal end	with 15–25 sharp, simple or rarely bifid teeth and 1–2 small spinules in middle part	with 17–20 teeth, upper with often blunt tips, lower smaller, with spiniform tips	terminates with four outgrowths
*P. indica*	3.4–3.3–2.8	roughly spherical, lacking lumpy inflations and radial ridges	large simple or bifid spines ventrally, smaller papillae laterally and ventrally	with terminal setae/spines	anterior lobes reduced, distinct rounded posterior rami	long setiform, with simple distal end	with speckled small denticles	feeble, uneven, with rare small denticles	with simple tip (not studied in details)
*P. madreporae*	2.9–1.5–1.4	elongated, with two developed posterior lobes	large spines ventrally, entire carapace with simple, rounded papillae	with minutel terminal spines	anterior lobes reduced, small, rounded posterior rami	not found	with 4 delicate multifid teeth	sclerotized, unarmed	with simple tip
*P. morula*	2.7–2.8–2.4	dome–shaped/spherical, with lumpy inflations, without radial ridges	large conical papillae ventrally, small papillae laterally	with small terminal spines	anterior lobes reduced, small, rounded posterior rami	long setiform, with simple distal end	with 10–14 sharp, simple teeth	with small denticles	terminates with two outgrowths
*P. nozawai*	4.2–3.3–3.6	roughly ovoid, with lumpy inflations, without radial ridges	numerous, large conical papillae ventrally, small papillae laterally	without terminal setae/spines	anterior lobes reduced, small, flat, rounded posterior rami	long setiform, with simple distal end	with 15 sharp, simple teeth	with 13–16 teeth, teeth in upper half with blunt tips, teeth in lower half smaller, spiniform	terminates with two outgrowths
*P. okadai*	1.35–?–0.77	dome–shaped, lacking lumpy inflations and radial ridges	few moderate papillae ventrally, small papillae scattered evenly over carapace	with terminal setae/spines	anterior lobes reduced, small, trapezoidal posterior rami	long setiform, with simple distal end	with 6–10 strong, slightly curved teeth, 1–2 lower teeth bifid or multifid	with ~ 10 weak, distally bifid or multifid teeth flanked by irregular row of 10–20 spinules	with simple tip (not studied in details)
*P. rubus*	5.71–?–5.65	Carapace roughly ovoid, with radial ridges with lumpy inflations	numerous, large conical papillae ventrally, small papillae laterally	without terminal setae/spines	anterior lobes reduced, small, rounded cylindrical posterior rami	long setiform, with simple distal end	with ~ 20 sharp, simple or rarely bifid teeth	with ~27 teeth, teeth in upper half with blunt tips, teeth in lower half smaller, irregular, spiniform	terminates with three long outgrowths and rudimentary subterminal seta
*P. sensoria*	1.6–1.4.–1.3	dome–shaped/spherical, lacking lumpy inflations and radial ridges	large, blunt papillae ventrally, small papillae elsewhere below midheight	with terminal setae/spines	anterior lobes reduced, short, broad, rounded posterior rami	long setiform, with simple distal end	with 7–8 upper recurved, simple teeth, and 2 lower bifid teeth	with ~ 9 distinct teeth, distal ones rounded, basal ones subdivided	with simple tip (not studied in details)
*Petrarca* sp. Grygier, 1991	1.5–?–1.1 (no holotype)	subglobular, lacking lumpy inflations and radial ridges	large blunt papillae and spines ventrally, small papillae laterally	without terminal setae/spines	anterior lobes reduced, small, rounded posterior rami	short setiform process on lobelike base	with 16 teeth, mostly bifid or trifid	with 12–14 teeth, tips mostly multifid	not studied in details

**Table 2.** Distribution and host records of Scleractinia infesting Ascothoracida (Petrarcidae). * – uncertain identification, hosts or dubious location; ? – unidentified petrarcid galls found in the same species of coral host.

Species	Locality	Coordinates	Depths, m	Host	Host taxonomy	Reference
1. *Petrarca azorica*	Azores	37°26'N, 25°52'W	835–1000	*Enallopsammia rostrata* (Pourtalès, 1878)	Refertina, Dendrophylliidae	Grygier 1985
2. *P. bathyactidis*	Pacific Ocean (~1200 km east of Japan)	35°41'N, 157°42'E	4200	Fungiacyathus (Bathyactis) symmetricus (Pourtalès, 1871) but really in *F. marenzelleri* (Vaughan, 1906)	Refertina, Fungiacyathidae	Fowler 1889; Grygier 1985
						Grygier 1981, 1983c
3. *P. chengi*	Dongsha Island, Taiwan;	20°33'40.8"N, 116°37'12.7"E	528	*Deltocyathus magnificus* Moseley, 1876	Vacatina, Deltocyathidae	herein
	Western Australia (?);	13°34'S, 122°54'E	390–394			Grygier 1991a
	Shikoku, Japan (?)	32°40'10"N, 132°25'15"E	393			Grygier and Nojima 1995
4. *P. goanna*	Lizard Island, Australia;	14°40'50"S, 145°27'39"E	2	*Turbinaria reniformis* Bernard, 1896	Refertina, Dendrophylliidae	Grygier 1991a
	Green Island, Taiwan	22°40'39.2"N, 121°28'57.2"E	4–20	*T. mesenterina* (Lamarck, 1816)		Kolbasov et al. 2023a
	Koh Khai Island, Thailand	10°41'56.5"N, 99°24'28.4"E		*T. frondens* (Dana, 1846)		
5. *P. indica*	South India	8°11'N, 74°03'E	1035	Flabellum (Ulocyathus) deludens Marenzeller, 1904	Refertina, Flabellidae	Grygier 1985
6. *P. madreporae*	Banda Sea, Indonesia	5°15S–22'S, 132°30'E–133°00'E	361–604	*Madrepora oculata* Linnaeus, 1758	Vacatina, Madreporidae	Grygier and Cairns 1996
	Chiba, Japan		480			Tachikawa et al. 2020
7. *P. morula*	Banda Neira Island, Indonesia;	4°35'S, 129°52'E		*Turbinaria* sp.	Refertina, Dendrophylliidae	Grygier 1985
	Amakusa, Okinawa, Japan;		10	*T. ? reniformis*, *T. frondens* (Dana, 1846),		Grygier and Nojima 1995
	Green Island, Taiwan	22°40'39.2"N, 121°28'57.2"E	4–20	*T. stellulata* (Lamarck, 1816),		Tachikawa et al. 2020
				*T. bifrons* Brüggemann, 1877		Kolbasov et al. 2023a
8. *P. nozawai*	Green Island, Taiwan	22°40'39.2"N, 121°28'57.2"E	4–20	*Turbinaria mesenterina* (Lamarck, 1816)	Refertina, Dendrophylliidae	Kolbasov et al. 2023a
	Keelung, Taiwan	25°7'49"N, 121°51'26"E	4–10	*Turbinaria* sp.		
9. *P. okadai*	Lizard Island, Australia;	14°39'S, 145°27'E	12–18	*Heteropsammia cochlea* (Spengler, 1781)	Refertina, Dendrophylliidae	Grygier 1981; Grygier 1991a
	Straits of Macassar, Indonesia	2°37.74'S, 117°49.41'E	45			
	Somalia*	11°14'N, 51°08'E	27–31	*Heteropsammia* sp.		Grygier 1985
*Petrarca* sp. (cf. *P. okadai*)*	Tanabe Bay, Japan*			*Tubastrea coccinea* Lesson, 1830		Grygier and Nojima 1995
10. *P. rubus*	Green Island, Taiwan	22°40'39.2"N, 121°28'57.2"E	4–20	*Turbinaria bifrons* Brüggemann, 1877	Refertina, Dendrophylliidae	Kolbasov et al. 2023a
				*Turbinaria* sp.		
11. *P. sensoria*	Queensland, Australia	27°31'S, 153°40'E	77–81	*Fungiacyathus* sp.	Refertina, Fungiacyathidae	Grygier 1991a
12. *Petrarca* sp. Grygier, 1991	Queensland, Australia	23°33.7'S, 152°37'E	270–348	*Anthemiphyllia dentata* (Alcock, 1902)	Vacatina, Anthemiphylliidae	Grygier 1991a
		26°32'S, 153°50'E				
		26°27'S, 153°50'E				
13. *Introcornia australis*	Saint Paul Island, southern Indian Ocean	38°48.8'S, 77°35.7'E	460–510	*Desmophyllum pertusum* (Linnaeus, 1758)	Vacatina, Caryophylliidae	Grygier 1991a
14. *I. conjugans*	Shikoku, Japan		100–300	Caryophyllia (Caryophyllia) quadragenaria Alcock, 1902	Vacatina, Caryophylliidae	Grygier 1983c
15. *Zibrowia auriculata*	Somalia;	11°11'N, 51°14'E	47–49	Balanophyllia (Eupsammia) carinata (Semper, 1872)	Refertina, Dendrophylliidae	Grygier 1985
	Kenya;	4°05'S, 39°41.9'E	30	*Tubastraea micranthus* (Ehrenberg, 1834)		
Z. ? reniformis*	Réunion;	12°52.1'S, 45°16.2'E	3–6	*Dendrophyllia* sp.		Grygier and Nojima 1995
	Comoro Islands;		30	*Tubastrea coccinea* Lesson, 1830		Tachikawa et al. 2020
	Tanabe Bay, Japan*		2–4	*T. micranthus*; *Dendrophyllia* spp.		
16. *Z. caudata*	Green Island, Taiwan	22°40'45.0"N, 121°30'12.0"E	4–20	*Tubastraea* sp.	Refertina, Dendrophylliidae	Kolbasov et al. 2025
17. *Z. mucronata*	Green Island, Taiwan	22°40'45.0"N, 121°30'12.0"E	20	*Tubastraea* sp.	Refertina, Dendrophylliidae	Kolbasov et al. 2025
18. *Z. trifurcata*	Green Island, Taiwan	22°40'45.0"N, 121°30'12.0"E	4–20	*Tubastraea* sp.	Refertina, Dendrophylliidae	Kolbasov et al. 2025
		22°41'27.7"N, 121°29'39.7"E				

## Material and methods

Five specimens of *Petrarca* sp. were collected from external galls on the deepwater coral *Deltocyathus magnificus* by Dr Yu-Rong Cheng (National Kaohsiung University of Science and Technology (**NKUST**), Taiwan), in the vicinity of Dongsha Island, Taiwan, at 528 m depth and fixed in 95% alcohol. Specimens of *Petrarca* were studied using light and scanning electron microscopy. For light microscopy, to make chitinous structures clearer, the holotype was treated with a 20% lactic acid water solution for 2–3 h. The carapace of the treated holotype was then removed to observe the body morphology. Carapace valves, trunk, dissected antennules, mouthparts, thoracopods, and furcal rami (Figs 2, 3) were mounted in glycerol on glass slide and examined using a LNZ-93B light microscope. Line drawings were made using a drawing tube on the same microscope.

For SEM examination of intact and dissected specimens (2), specimens were post-fixed in 2% OsO_4_ for 2 h, dehydrated in acetone, and critical-point dried from CO_2_ in a Hitachi HCP-2 critical point dryer. Dried specimens were sputter-coated with platinum–palladium in an Eiko IB-3 Ion Coater and examined by GAK in a JEOL JSM-6380LA scanning electron microscope at Moscow State University. The resulting photographs were touched up using CorelDraw X3 Graphics Suite, which was also used to prepare all the figures presented here.

## Results

### Systematics

**Subclass Ascothoracida Lacaze-Duthiers, 1880**

#### Order Laurida Grygier, 1987

**Family Petrarcidae Gruvel, 1905**

##### Genus Petrarca

Taxon classificationAnimaliaLauridaPetrarcidae

Fowler, 1889

34734950-95DD-5177-AB57-CE16A2F9B8A4

###### Type species.

*Petrarca bathyactidis* Fowler, 1889

##### Petrarca chengi sp. nov.

Taxon classificationAnimaliaLauridaPetrarcidae

C92046B5-7BD7-58FE-B497-FBE4B1C63AA1

https://zoobank.org/B83DB387-56DD-460C-9FB8-7AAC2D0F3C7B

**Figure 1.**
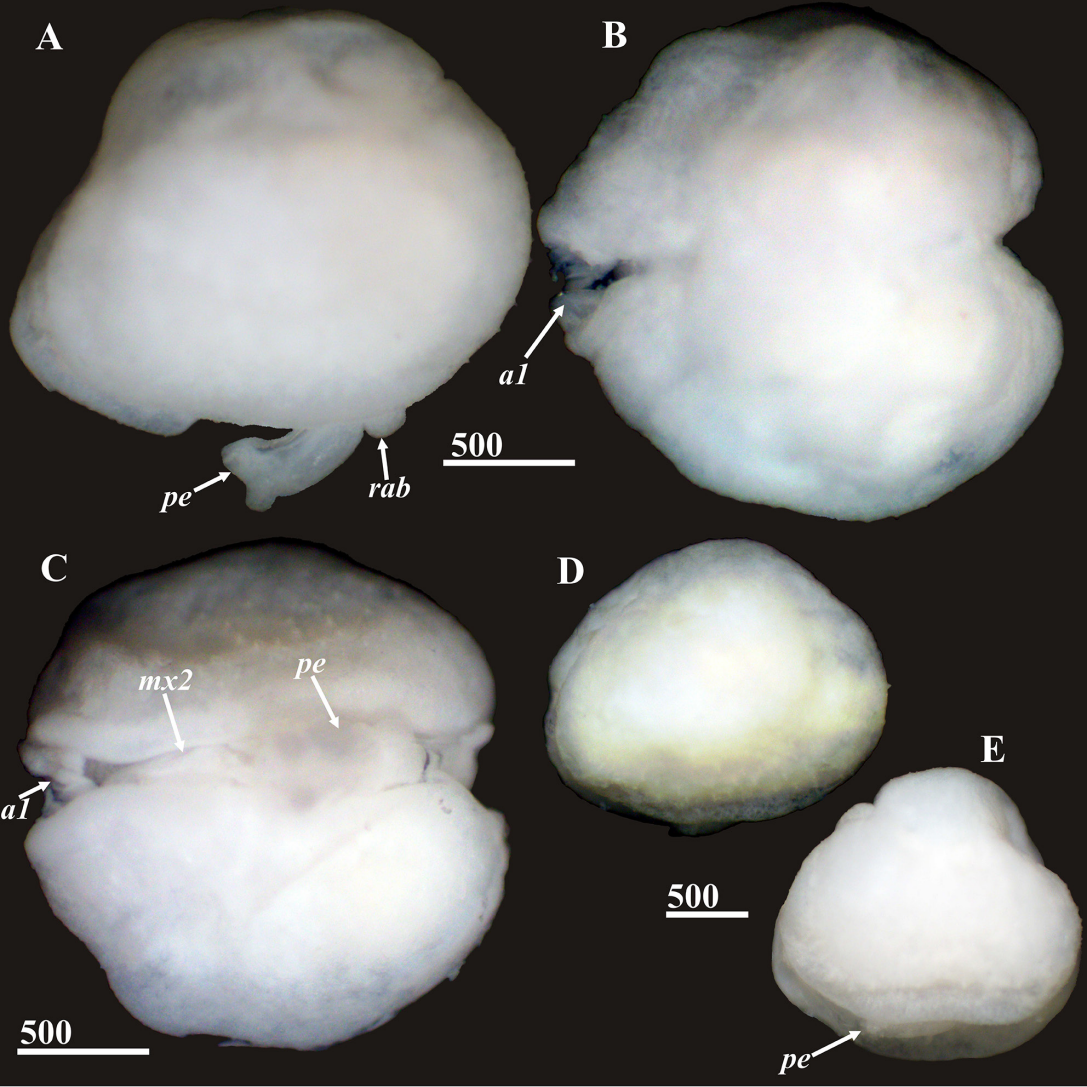
*Petrarca chengi* sp. nov., habitus (after alcohol fixation), anterior end left, light microscopy (**A–C**. Holotype; **D, E**. Paratypes). **A**. Lateral view, left side; **B**. Dorsal view; **C**. Ventral view; **D, E**. Lateral view, left side. Abbreviations: *a1* = antennules, *mx2* = maxillae, *pe* = penis, *rab* = rudimentary abdomen. Scale bars in µm.

**Figure 2.**
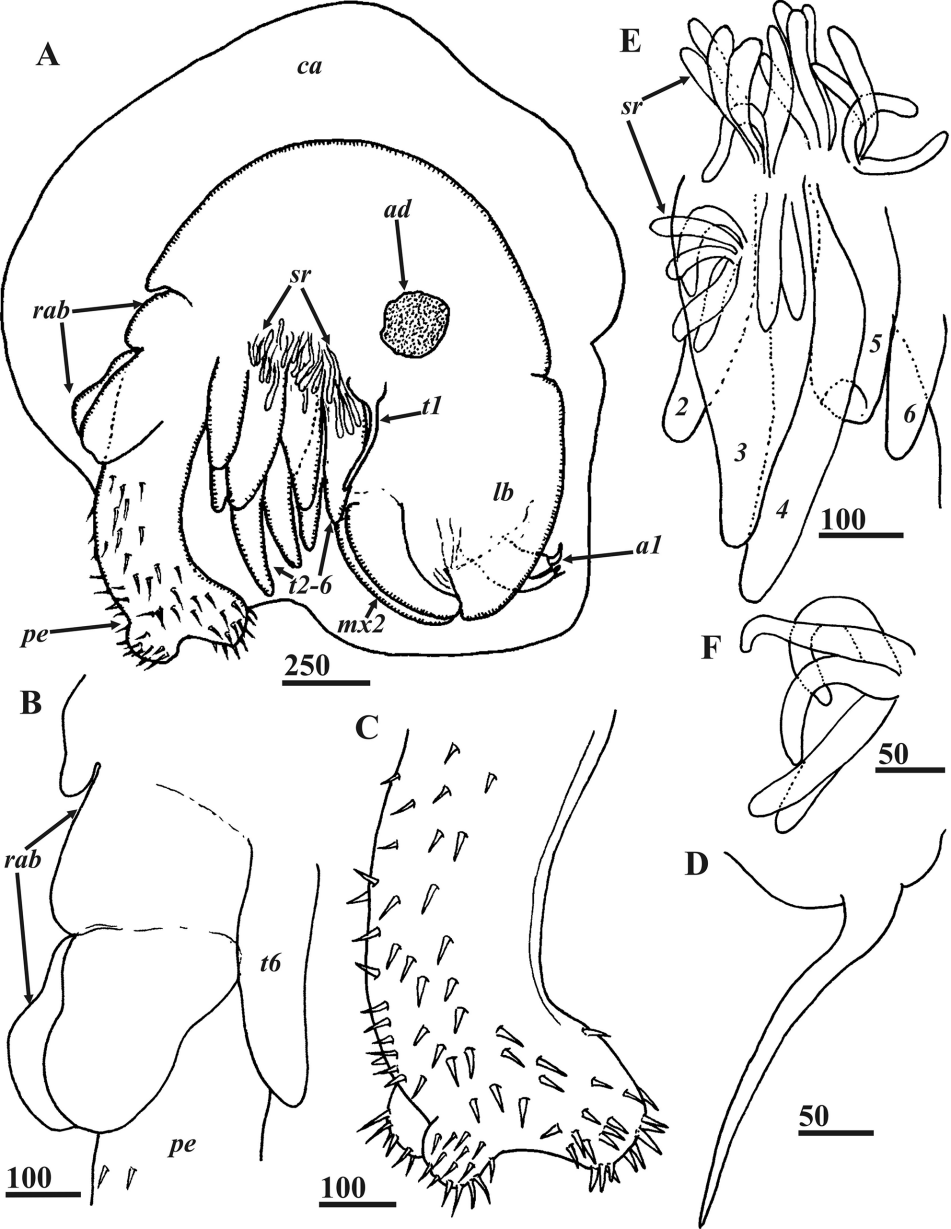
*Petrarca chengi* sp. nov., holotype, female, light microscopy. General morphology. **A**. Lateral view, right side with body proper (right valve of carapace removed); **B**. Rudimentary abdomen; **C**. Distal half of penis; **D**. Thoracopod 1; **E**. Thoracopods 2–6 (numbered) with seminal receptacles; **F**. Seminal receptacles of thoracopod 2. Abbreviations: *a1* = antennule, *ad* = adductor muscle, *ca* = carapace, *lb* = labrum, *mx2* = maxillae, *pe* = penis, *rab* = rudimentary abdomen, *sr* = seminal receptacles, *t1-6* = thoracopods. Scale bars in µm.

**Figure 3.**
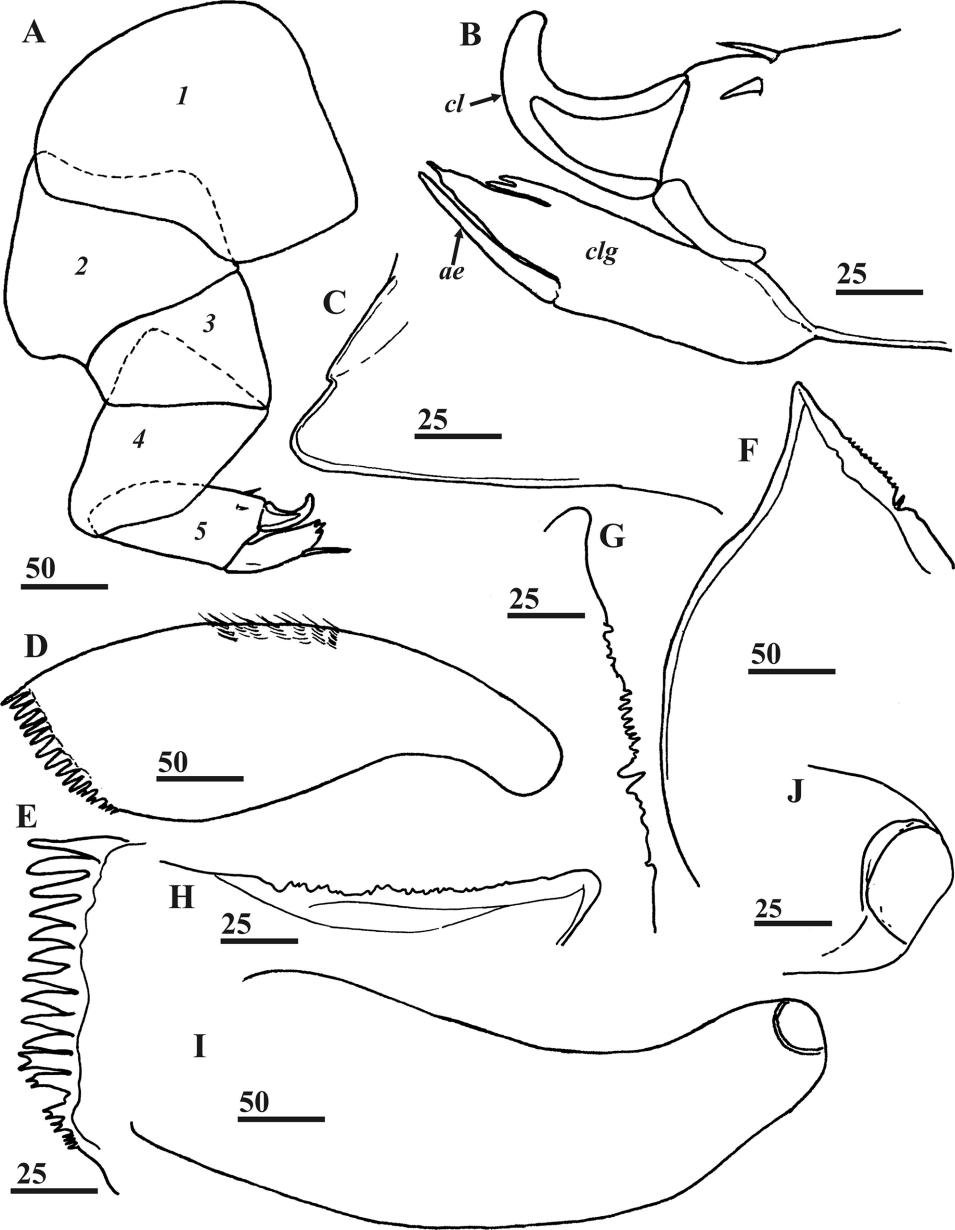
*Petrarca chengi* sp. nov., holotype, female, light microscopy. Antennules and mouth parts. **A**. Antennule, segments numbered; **B**. Claw and claw guard of last (5^th^) antennular segment; **C**. Medial languette; **D**. Mandible; **E**. Cutting edge of mandible; **F**. Maxillule; **G, H**. Cutting edges of maxillules; **I**. Maxilla; **J**. Tip of maxilla. Abbreviations: *ae* = aesthetasc, *cl* = claw, *clg* = claw guard. Scale bars in µm.

**Figure 4.**
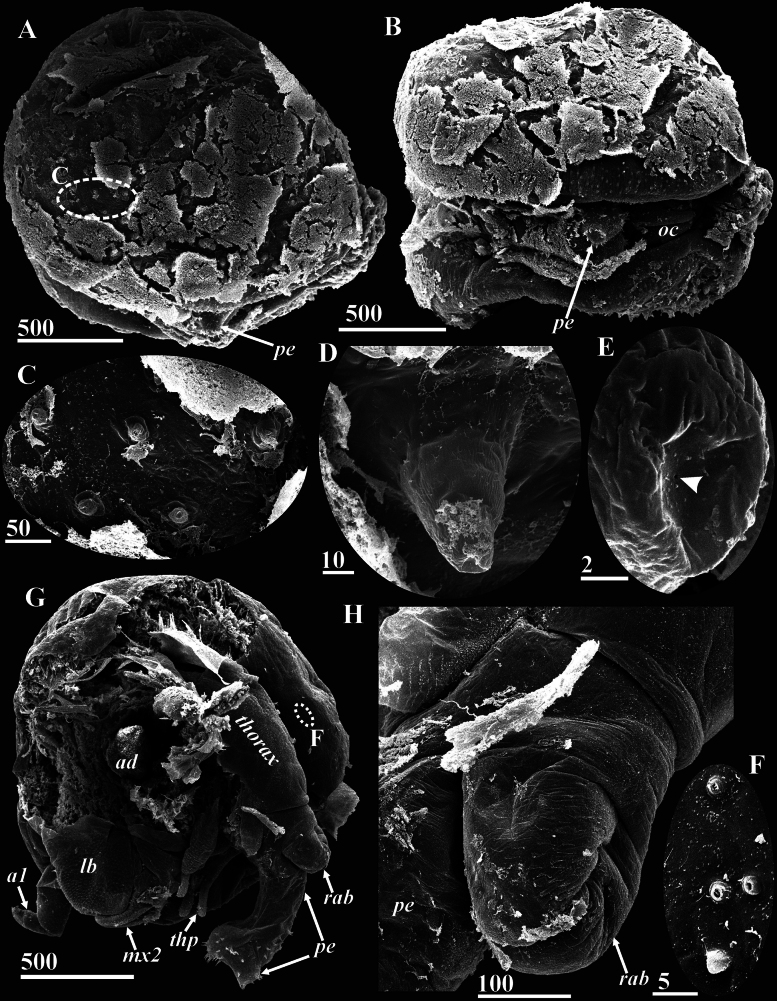
*Petrarca chengi* sp. nov., general view and mantle (carapace) structures (SEM). **A**. Habitus, lateral view, right side; **B**. Habitus, ventrolateral view; **C**. External surface of mantle with papillae, central part; **D**. External papilla of mantle; **E**. Tip of papilla with micro-pore (indicated by arrowhead). **F**. Micro-pores on surface of internal cuticle of mantle; **G**. Habitus with left valve removed; **H**. Rudimental abdomen, dorsolateral view. Abbreviations: *a1* = antennule, *ad* = adductor muscle, *lb* = labrum, *mx2* = maxillae, *oc* = oral cone, *pe* = penis, *rab* = rudimentary abdomen, *thp* = thoracopods. Scale bars in µm.

**Figure 5.**
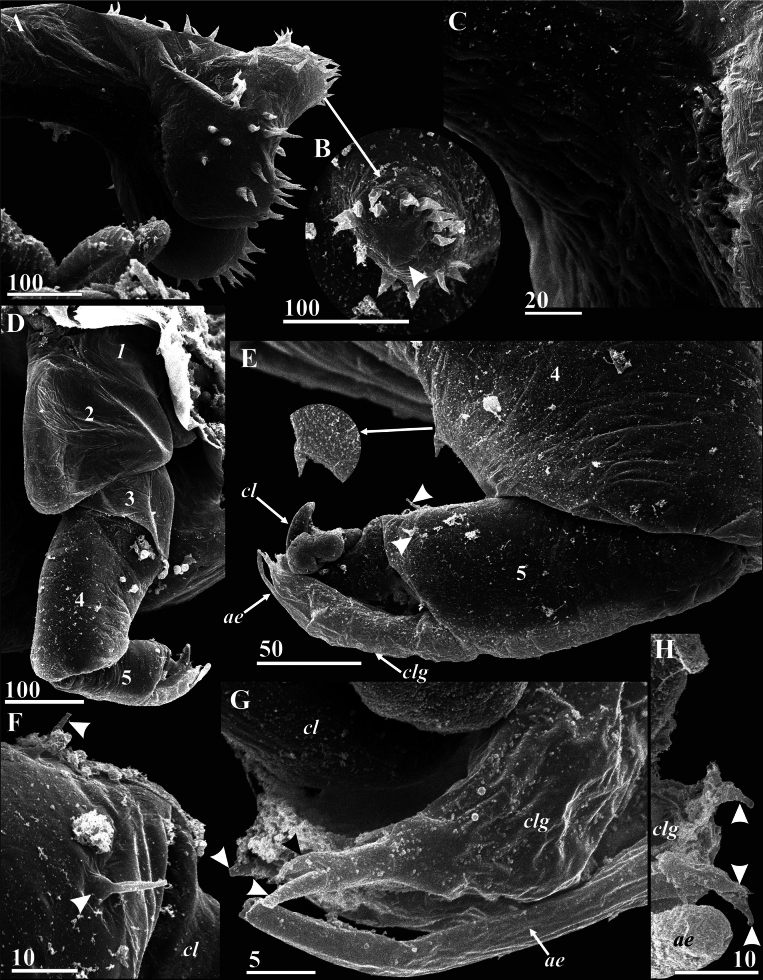
*Petrarca chengi* sp. nov., penis and antennules (SEM). **A**. Distal part of penis, anterolateral view; **B**. Posteroterminal process of penis with slit opening (indicated by arrowhead); **C**. Sponge-like cuticle on anterior part of penis; **D**. Left antennule, general view, segments numbered; **E**. Distal part of right antennule, segments numbered, rudimentary seta on 4^th^ segment enlarged, tiny setae at base of claw indicated by arrowheads; **F**. Tiny setae at base of claw (indicated by arrowheads), left antennule; **G**. Distal part of claw guard, 3 rudimentary setae indicated by arrowheads, right antennule; **H**. Tip of claw guard, 3 rudimentary setae indicated by arrowheads, left antennule. Abbreviations: *ae* = aesthetasc, *cl* = claw, *clg* = claw guard. Scale bars in µm.

**Figure 6.**
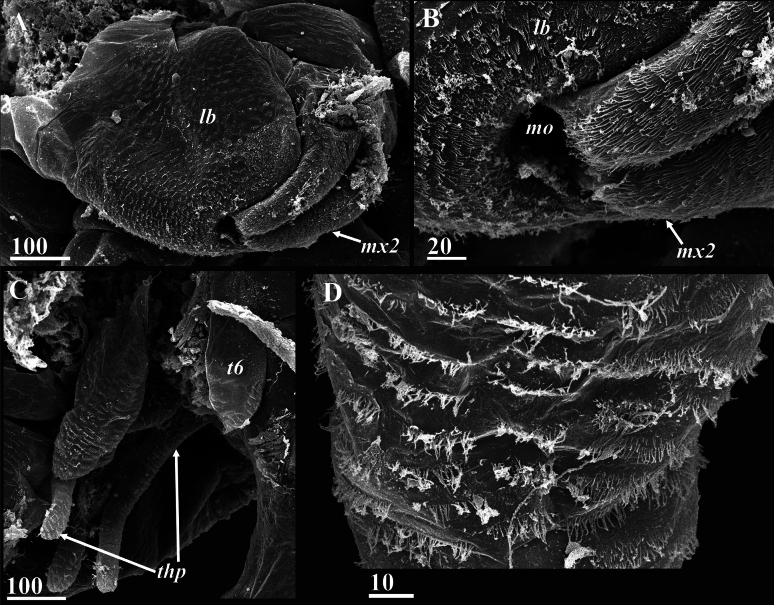
*Petrarca chengi* sp. nov., oral cone and thoracopods (SEM). **A**. Oral cone, ventrolateral view; **B**. Mouth opening; **C**. Thoracopods; **D**. Ctenoid scales on thoracopod 3. Abbreviations: *lb* = labrum, *mo* = mouth opening, *mx2* = maxillae, *t6* = thoracopod 6, *thp* = thoracopods 2-5.

Figs 1, 2, 3, 4, 5, 6

###### Material examined.

• Five specimens (including holotype) in *Deltocyathus magnificus* Moseley, 1876, 20°33'40.8"N, 116°37'12.7"E, Dongsha Island, Taiwan, 30.07.2015, 528 m depth, station CP4162, coll. Dr Cheng Yu-Rong. Glycerin slide of the holotype (no. Mg 1270) with the dissected mouth parts, antennules, and rest of the body proper with thoracopods and penis, and an undissected paratype (no. Mg. 1271) in 95% alcohol, are deposited in the Zoological Museum of Moscow State University in Moscow, Russian Federation. One undissected paratype, preserved in 95% alcohol, is deposited in the Biodiversity Research Museum, Biodiversity Research Center, Academia Sinica, Taipei, Taiwan (ASIZCR-000495). The stub with two specimens studied with SEM were kept for further investigation (in the collection of GAK).

###### Diagnosis.

Carapace roughly dome-shaped; valves without lumpy inflations and radial ridges; carapace margins not crenulated; ventral side of carapace with numerous small papillae; lateral surface with large, conical, volcano-like papillae. Antennular claw guard with simple aesthetasc. Mandible with 10 upper long, sharp, simple teeth and 4 lower bifid teeth; lower angle with tuft of 4 small, sharp denticles; maxillules with uneven, saw-like cutting margin; 6 pairs of thoracopods; first thoracopod setiform; penis with conspicuous posterior cylindrical rami with corona of short, wide setae.

###### Etymology.

The species is named after Dr Yu-Rong Cheng, our colleague, who found specimens of this species.

###### Description.

Adult (mature) specimens 1.8–2.0 mm long, 1.5–1.8 mm high, and 1.5–1.9 mm wide (Figs 1, 4A, B). ***Carapace*** (Figs 1, 4A, B) roughly dome-shaped, slightly narrowing towards anterior end; valves without lumpy inflations, ridges, or other macrosculpture; margins of carapace rounded, not crenulated; ventral side of carapace with numerous small papillae; ventrolateral surface with large, conical, volcano-like papillae, 60–70 µm high (Fig. 4A–E). Carapace papillae terminating at central microscopic pore (Fig. 4E). Internal cuticle of carapace (mantle) with scattered micro-pores (Fig. 4F). ***Body*** inflated, curved, enclosed between carapace valves; tip of penis close to oral cone and protruding outward (Figs 1A, 1C, 1E, 2A, 4A, 4B, 4G). ***Cephalon*** with large adductor muscle lying above big oral cone flanked by 5-segmented antennules. ***Thorax*** with sinusoid arched dorsal margin, without distinct segmentation, with cluster of rudimentary uniramous thoracopods (Figs 2A, 4G). ***Abdomen*** with conspicuous first segment bearing long penis; posterior part rudimentary, with distinct cleft, bilobed, about equal in length to first segment, without terminal spines and setae (Figs 2A, 2B, 4G, 4H).

***Antennules*** somewhat W-shaped and prehensile, with little armament of external sculpture on 2 distal segments (Figs 3A, 3B, 5D–H). First and second segments irregularly trapezoidal, narrowing toward upper/dorsal margin, with slightly curved ventral/postaxial margin; third segment trapezoidal, narrowing toward lower/ventral margin; fourth segment longer than wide, narrowing distally, with almost straight ventral and dorsal margins, thick, short subdistal seta inserted at anteriodorsal corner (Fig. 5E). Fifth segment rectangular, slightly narrowing towards proximal end, shorter and narrower than fourth, and armed with sensory and grasping structures (Figs 3A, 3B, 5E–H). Short, massive, curved movable claw with smooth, concave margin arising from distal end of segment. Three rudimentary setae at base of claw and to each side (Figs 3B, 5E, 5F); lateral sides without pores. ***Claw*** sheathed by large, hood-shaped claw guard on posterodistal corner; claw guard with 3 vestigial, distal setae with terminal pore; developed subterminal aesthetasc half as long as claw guard, terminates with simple end without outgrowths (Figs 3B, 5G, 5H).

***Oral cone*** prominent, consisting of massive labrum posteriorly underlaid with massive, fused maxillae, unpaired medial languette, and paired mandibles and maxillules (Figs 2A, 3C–J, 4G, 6A, 6B). Massive prow-shaped labrum with short posterolateral extensions, leaving maxillae largely exposed, dense ctenoid scales on the exterior (Figs 2A, 4G, 6A, 6B). ***Mandibles*** (Fig. 3D, F) elongated, outer/upper margin with transverse rows of dense, thin setae in middle; cutting edge straight, with 10 upper long, sharp, simple teeth and 4 lower bifid teeth; lower angle with tuft of 4 small, sharp denticles. ***Maxillules*** (Fig. 3F–H) with sclerotized, triangular distal parts, with uneven, saw-like, cutting inner margin. Fused ***maxillae*** (Figs 3I, 3J, 6A, 6B) with dense ctenoid scales on lateral and ventral surfaces; distal ends with rounded zones of sclerotized, thick, wrinkled cuticle without denticles, pores, or setules.

Six pairs of ***thoracopods***; thoracopods 2–6 uniramous, unsegmented, grouped in cluster, and arranged in an unorderly sequence in lateral view (Figs 2A, 2E, 4G, 6C). Thoracopod 1 setiform (Fig. 2A, D); thoracopods 2–4 conical, longer and wider than thoracopods 5, 6; thoracopod 6 smaller, 2–3 times shorter than others. Batteries of ampuliform seminal receptacles (Fig. 2E, F) associated with thoracopods 2–5 (5, 8–9, 4–5 and 4 receptacles, respectively). Cuticle of thoracopods 2–6 with dense, long ctenoid scales (Fig. 6D).

Long (~0.7–0.8 mm) and massive, terminally bifid ***penis*** originating from large first abdominal segment (Figs 1A, 2A, 2C, 4G, 5A). Shaft of penis supports two developed, rounded anterior lobes and two conspicuous posterior cylindrical-rectangular rami about 80–90 µm long, with corona of short, wide setae and slit-like terminal opening (Figs 2C, 4G, 5A, 5B). Distal half of penis, including rami, bearing numerous short but wide conical setae; anterior cuticle with “sponge”-like structure (Figs 2C, 5A, 5C).

###### Remarks.

*Petrarca chengi* sp. nov. resembles *P. azorica* Grygier, 1985, *P. bathyactidis* Fowler, 1889, *P. indica* Grygier, 1985, *P. okadai* Grygier, 1981, *P. sensoria* Grygier, 1991, and *Petrarca* sp. Grygier, 1991 in having a dome-shaped to subspherical or ellipsoid carapace lacking lumpy inflations and radial ridges on the valves (Table 1). The new species differs from *P. azorica*, *P. bathyactidis*, *P. indica*, and *Petrarca* sp. Grygier, 1991 by the absence of bumps and large, irregular projections or bifid spines along the ventral margin of the carapace valves. In comparison with *P. okadai*, *P. chengi* sp. nov. possesses larger lateral papillae on the carapace. *Petrarca chengi* sp. nov. can also be distinguished from *P. azorica*, *P. indica*, *P. okadai*, and *P. sensoria* by the absence of terminal setae or spines on the rudimentary abdomen and by having fewer but larger mandibular teeth (a condition similar to that of *P. sensoria*). The maxillule of the new species bears an uneven, saw-like inner cutting margin, lacking the well-developed multifid teeth observed in *P. okadai*, *P. sensoria*, and *Petrarca* sp. Grygier, 1991. The first setiform thoracopod of *P. chengi* sp. nov. terminates in a simple distal end, contrasting with the two distal setae found in *P. bathyactidis*. The penis bears two well-developed, rounded anterior lobes, which are absent in *P. okadai*, *P. sensoria*, and *Petrarca* sp. Grygier, 1991.

**Figure 7.**
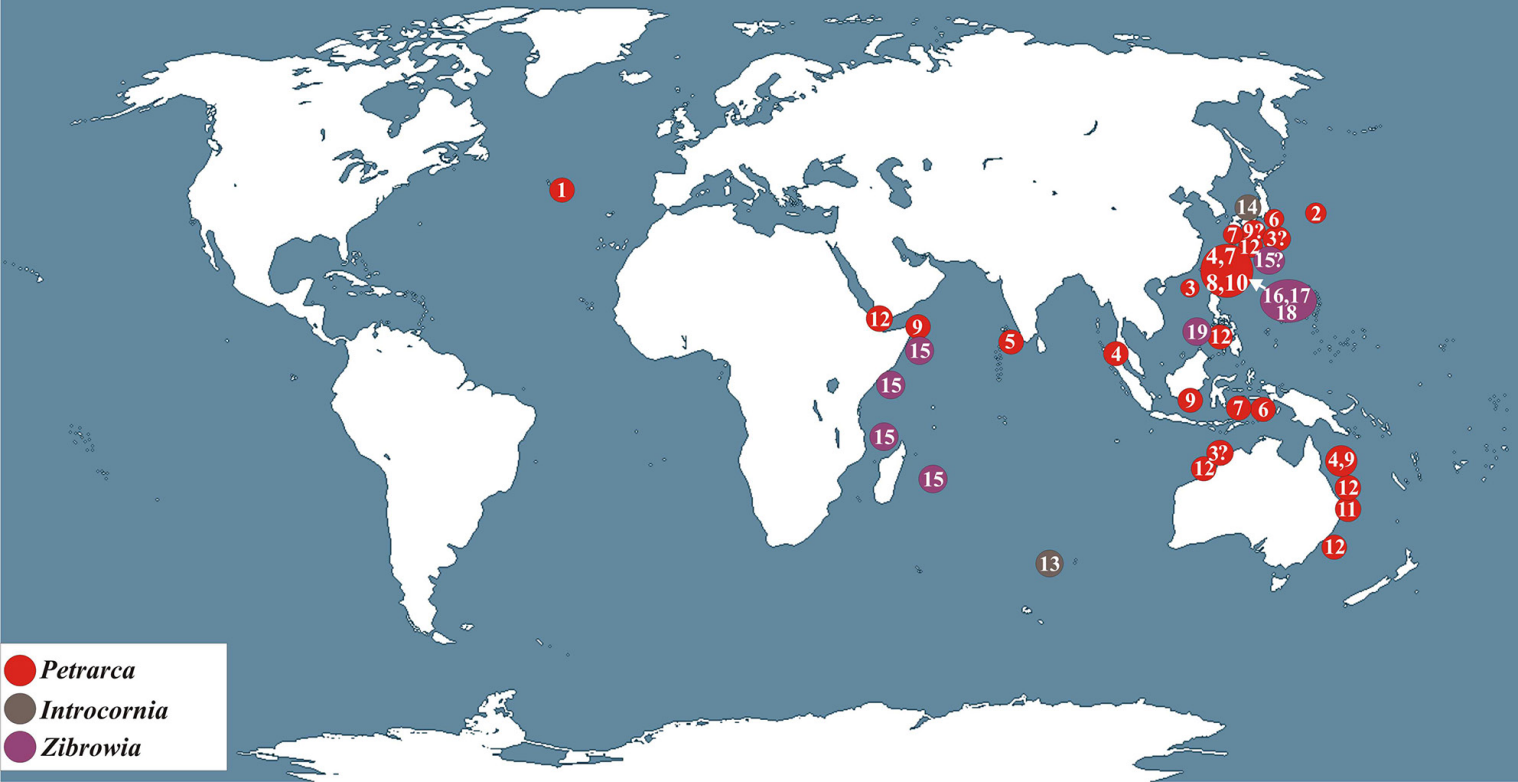
Map of distribution records of Ascothoracida of family Petrarcidae (? = doubtful record, see text for explanation). 1. *Petrarca azorica* Grygier, 1985; 2. *Petrarca bathyactidis* Fowler; 3. *Petrarca chengi* sp. nov.; 4. *Petrarca goanna* Grygier, 1991; 5. *Petrarca indica* Grygier, 1985; 6. *Petrarca madreporae* Grygier, 1996; 7. *Petrarca morula* Grygier, 1985; 8. *Petrarca nozawai* Kolbasov, Savchenko, Yu, Tsao, Ganmanee & Chan, 2023; 9. *Petrarca okadai* Grygier, 1981; 10. *Petrarca rubus* Kolbasov, Savchenko, Yu, Tsao, Ganmanee & Chan, 2023; 11. *Petrarca sensoria* Grygier, 1991; 12. Unidentified *Petrarca* specimens; 13. *Introcornia australis* Grygier, 1990; 14. *Introcornia conjugans* Grygier, 1983; 15. *Zibrowia auriculata* Grygier, 1985; 16. *Zibrowia caudata* Kolbasov, Tsao & Chan, 2025; 17. *Zibrowia mucronata* Kolbasov, Tsao & Chan, 2025; 18. *Zibrowia trifurcata* Kolbasov, Tsao & Chan, 2025; 19. Unidentified *Zibrowia* specimens.

Dry, indefinite petrarcid galls have previously been reported on the same coral species, *Deltocyathus magnificus*, from Western Australia and southern Japan (Grygier 1991a; Grygier and Nojima 1995). We presume that these galls also belong to *P. chengi* sp. nov. (Fig. 7).

## Discussion

### Diversity and morphology of the genus Petrarca

Taxonomically, ascothoracidans are distinguished by several key morphological characters, including the structure and shape of the carapace, the morphology of the trunk somites, furcal rami, thoracopods, penis, antennules, and mouthparts. Carapace morphology constitutes one of the most taxonomically significant diagnostic features within the genus *Petrarca* (Kolbasov et al. 2023a). Recently, Kolbasov et al. (2023a) provided a comprehensive morphological analysis of four species of *Petrarca* from Taiwan, including a detailed examination of their ultrastructure and suggested that the monophyly of the genus could be established based on the fine morphological details revealed with SEM. Congeners of *Petrarca* can be distinguished by the following morphological features: (i) the form of the carapace, (ii) the morphology of carapace papillae and spines, (iii) the structure of the rudimentary abdomen, (iv) the shape of the distal part of the penis, (v) the morphology of thoracopod 1, (vi–vii) the cutting margins of the mandibles and maxillules, and (viii) the ultrastructure of the distal part the aesthetasc of antennular claw guard. All these diagnostic characters are summarized for all known species of *Petrarca* in Table 1.

Thus, species of *Petrarca* inhabiting *Turbinaria* corals have carapace valves with conspicuous lumpy bumps and a crenulated margin (*P. goanna*, *P. morula*, *P. nozawai*, *P. rubus*), while other congeners have more or less smooth carapace valves. The carapace in *Petrarca* is normally roughly spheroidal, ovoid, or dome-shaped. However, in *P. madreporae*, carapace valves have developed posterior lobes, a characteristic of another petrarcid genus, *Zibrowia*, so the likelihood of it being in this genus is high (Grygier and Cairns 1996; Kolbasov et al. 2023a; Table 1). Species of *Petrarca* are characterized by having numerous large, conical papillae on the ventral to anteroventral surface of the carapace, along with smaller papillae scattered across the entire lateral surface. The distribution pattern of these papillae varies among species (Table 1).

Three species (*P. chengi*, *P. nozawai*, *P. rubus*) and *Petrarca* sp. Grygier, 1991 lack terminal spines or setae on the rudimentary abdomen, whereas the remaining species possess them (Table 1). The morphology of the distal part of the penis varies among species. The anterior lobes may be reduced (*P. azorica*, *P. goanna*, *P. indica*, *P. madreporae*, *P. morula*, *P. nozawai*, *P. okadai*, *P. rubus*, *P. sensoria*, *Petrarca* sp. Grygier, 1991), small (*P. bathyactidis*), or well developed (*P. chengi*). The penis rami may be rectangular or square (*P. azorica*, *P. chengi*, *P. goanna*), triangular (*P. bathyactidis*, *P. okadai*), or rounded (*P. indica*, *P. madreporae*, *P. morula*, *P. nozawai*, *P. rubus*, *P. sensoria*).

Most species possess, when present, a long setiform thoracopod 1 with a simple distal end, whereas *P. bathyactidis* has a long setiform thoracopod 1 bearing two short distal setae, and *Petrarca* sp. Grygier, 1991 has a short setiform thoracopod 1 arising from a lobe-like base. The armament on the cutting edge of the mandible and maxillules also varies among species of the genus. The mandibles may bear only simple teeth (*P. morula*, *P. nozawai*), a combination of simple and bifid teeth (*P. chengi*, *P. goanna*, *P. okadai*, *P. rubus*, *P. sensoria*), or simple, bifid, and multifid teeth (*P. azorica*, *P. madreporae*). The cutting (inner) edge of the maxillules may be unarmed (*P. madreporae*), armed with small denticles (*P. chengi*, *P. indica*, *P. morula*), or equipped with blunt upper teeth and/or sharp multifid lower teeth (*P. azorica*, *P. goanna*, *P. okadai*, *P. nozawai*, *P. rubus*, *P. sensoria*). The morphology (ultrastructure) of the antennular aesthetasc located on the claw guard may also have high taxonomic value. This aesthetasc may represent a modified and reduced proximal sensory process of the antennules found in generalized ascothoracidans, including the basal petrarcid genus *Introcornia*. When described, it shows variation in the number of terminal outgrowths or setae: one in *P. chengi* and *P. madreporae*; two in *P. bathyactidis*, *P. morula* and *P. nozawai*; three in *P. rubus* and *P. okadai*; and four in *P. goanna*.

### Host specificity of the scleractinian-infesting Ascothoracida

The family Petrarcidae (genera *Introcornia*, *Petrarca*, *Zibrowia*) represents one of the most specialized ascothoracidans exclusively parasitizing corals of the order Scleractinia (Table 2). The petrarcids infest both scleractinian suborders, Refertina and Vacatina. The genus *Introcornia* (2 species) infests exclusively deep-water azooxanthellate Vacatina corals of the family Caryophylliidae (*Desmophyllum pertusum* (Linnaeus, 1758) and *Caryophyllia quadragenaria* Alcock, 1902). Whereas *Zibrowia* (4 species) has been found in shallow-water Refertina corals of the family Dendrophylliidae (Table 2). *Zibrowia* infests the solitary zooxanthellate coral *Balanophyllia carinata* (Semper, 1872) and azooxanthellate colonial corals of the genera *Dendrophyllia* and *Tubastraea*.

The genus *Petrarca* (11 valid species) parasitizes a broader range of scleractinians from both suborders, Refertina and Vacatina (Table 2). Six species (*P. azorica*, *P. goanna*, *P. morula*, *P. nozawai*, *P. okadai*, *P. rubus*) have been recorded from Refertina corals of the family Dendrophylliidae, including the zooxanthellate genus *Turbinaria* and the azooxanthellate genera *Enallopsammia*, *Heteropsammia*, and *Tubastraea*. Two species (*P. bathyactidis*, *P. sensoria*) infest Refertina azooxanthellate corals of the genus *Fungiacyathus* (Fungiacyathidae). *Petrarca chengi* has been described from the Vacatina azooxanthellate coral *Deltocyathus magnificus* (Deltocyathidae), *P. indica* from the Refertina azooxanthellate coral *Flabellum deludens* Marenzeller, 1904 (Flabellidae), and *P. madreporae* from the Vacatina azooxanthellate coral *Madrepora oculata* Linnaeus, 1758 (Madreporidae).

The fact that *P. madreporae*, whose taxonomic position is still uncertain (see earlier discussion), was originally described from Vacatina corals may support its placement in *Petrarca* rather than *Zibrowia*. Species of *Zibrowia* are known to inhabit only Refertina corals within the family Dendrophylliidae.

Recent studies have demonstrated that petrarcids are true endoparasites of corals and possess mandibles with a row of sharp and dense teeth to cut coral (holobiont) tissue unselectively (Kolbasov et al. 2023b; Zalota et al. 2025). Consequently, they are more or less evenly distributed among both azooxanthellate and zooxanthellate coral hosts.

### Geographical and vertical distribution of the scleractinian-infesting Ascothoracida

Petrarcidae are widespread in the tropical and subtropical waters of the Indo-West Pacific region, with the exception of *P. azorica*, which occurs in the Azores Islands (Fig. 7; Table 2). They extend from 37°N (Azores) and 35°N (Japan) in the Northern Hemisphere to 38°S (Saint Paul Island, southern Indian Ocean), 34°S (Australia) in the Southern Hemisphere (Table 2; Fig. 7). Five of the 17 valid species occur in the Indian Ocean, whereas the majority (13 species) has been described from the Pacific. On present knowledge, species of *Petrarca* are the most widespread within the family and occur in the Pacific (9 species), Indian (3 species), and Atlantic (1 species) oceans. In addition, numerous unidentified *Petrarca* specimens have been recorded from various localities across the Indo-West Pacific. The occurrence of *Petrarca* in the North Atlantic may indicate a wider distribution of petrarcids in the Atlantic Ocean. Petrarcidae exhibit a Tethyan relict pattern in their distribution (Fig. 7).

The vertical distribution of six *Petrarca* species (*P. goanna*, *P. morula*, *P. nozawai*, *P. okadai*, *P. rubus*, and *P. sensoria*) extends from the upper to the lower subtidal zone. Four species (*P. azorica*, *P. chengi*, *P. indica*, *P. madreporae*) have been recorded from the bathyal zone, while only *P. bathyactidis* has been found in the abyssal zone (Table 2). All four species of *Zibrowia* inhabit the upper subtidal zone, whereas the two species of *Introcornia* occur in the lower subtidal to upper bathyal zones (Table 2).

## Supplementary Material

XML Treatment for Petrarca

XML Treatment for Petrarca chengi

## Additional information

### Artificial Intelligence (AI) use

The authors accept full responsibility for the content of the manuscript, including the disclosure of any use of AI.

No AI tools were used in the preparation of this manuscript.

### Author ORCIDs

G.A. Kolbasov https://orcid.org/0000-0002-3762-1834

B.K.K. Chan https://orcid.org/0000-0001-9479-024X

### Data availability

Holotypes and paratypes were deposited in the Zoological Museum of Moscow State University, Moscow, Russian Federation and Biodiversity Research Museum, Academia Sinica, Taiwan.
